# Uretero-Iliac Artery Fistula: Intraoperative Diagnosis of a Rare but Fatal Cause of Hematuria

**DOI:** 10.7759/cureus.112505

**Published:** 2026-07-12

**Authors:** Ishan Jain, Naveen Kulasingha, Thamer Mhanna, Hani Sayedin, Rene Woderich

**Affiliations:** 1 Urology, North Cheshire and Mersey NHS Foundation Trust, Warrington, GBR

**Keywords:** endourology, pelvic irradiation, ureteric jj stent, uretero-iliac artery fistula, urinary tract trauma

## Abstract

Uretero-iliac artery fistula (UIAF) is a rare but often fatal type of fistula. We present the case of a 58-year-old woman who presented with recurrent hematuria. During her admission, she underwent a routine ureteric stent exchange. During the procedure, she developed sudden hemorrhagic shock during guidewire insertion, and a diagnosis of UIAF was suspected. The patient required resuscitation and urgent right common iliac artery stent placement by interventional radiology (IR) following discussion between vascular surgeons and the IR team. She was admitted to the intensive therapy unit following the IR intervention, where she recovered well without complications. She was discharged home three days later with outpatient vascular and urology follow-up. UIAF is a rare but important differential diagnosis of hematuria. A high index of clinical suspicion is important to facilitate its diagnosis. Eliciting the patient’s past medical history and risk factors is of paramount importance in raising suspicion for UIAF.

## Introduction

Uretero-iliac artery fistula (UIAF) occurs when an abnormal connection forms between the ureter and the iliac artery. This condition often poses a significant diagnostic challenge. CT angiography (CTA) is the gold standard initial imaging investigation but is often negative even in confirmed cases, with a reported sensitivity of 62%. CTA is a noninvasive, CT-based imaging technique that uses intravenous contrast to produce rapid, high-resolution three-dimensional images of blood vessels, whereas digital subtraction angiography is an invasive, catheter-based X-ray procedure that injects contrast directly into the artery and remains the gold standard for detailed vascular imaging [1].

UIAF is rare, with approximately 613 cases reported between 1908 and 2022 [2], and is associated with relatively high mortality rates of 7-23% [3]. Risk factors for UIAF include previous radiotherapy, abdominal trauma, vascular surgery, and ureteric stents. Due to the significant association with a history of pelvic surgery or irradiation, increasing diagnostic suspicion in the emergency setting should be considered preoperatively in such patients.

This report describes the first case of UIAF diagnosed at our hospital, in which the diagnosis was made intraoperatively using retrograde ureteropyelography performed during a ureteric stent exchange. This case highlights the importance of timely intraoperative identification of UIAF, as well as considering UIAF as a differential diagnosis in patients presenting to the emergency setting with recurrent hematuria and known risk factors for UIAF.

## Case presentation

We present the case of a 58-year-old woman with a history of recurrent hematuria. On this admission, the patient presented late at night with pain around her right nephrostomy site, which had been placed because of longstanding severe hydronephrosis caused by an encrusted ureteric stent. The stent had been inserted two years earlier but had become encrusted after the patient missed her follow-up appointment. The nephrostomy had been inserted one month before this admission to relieve the hydronephrosis caused by the encrusted stent. The ureteric stent had originally been inserted to bypass a right ureteric stricture that developed following multiple courses of radiotherapy administered for cervical cancer, which was successfully treated in 2004.

Given the history of radiotherapy and the encrusted stent, these were considered the most likely causes of her hematuria. The patient was scheduled for an elective ureteric stent exchange three days after admission. She reported dysuria but was otherwise systemically well, with no other lower urinary tract symptoms. On examination, her observations showed a heart rate of 120 beats/min, but she was otherwise hemodynamically stable. The patient remained hemodynamically stable throughout her admission. Three days before the elective procedure, her hemoglobin (Hb) level was 96 g/L, down from 103 g/L one week earlier. Before the procedure, her Hb gradually declined to 68 g/L (Table 1).

**Table 1:** Patient Hb level Hb, hemoglobin

Time point	Patient Hb value	Normal range
One week before the procedure	103 g/L	115-165 g/L
Day 1 (admission)	96 g/L	115-165 g/L
Day 4 (day of procedure)	68 g/L	115-165 g/L

**Figure 1:**
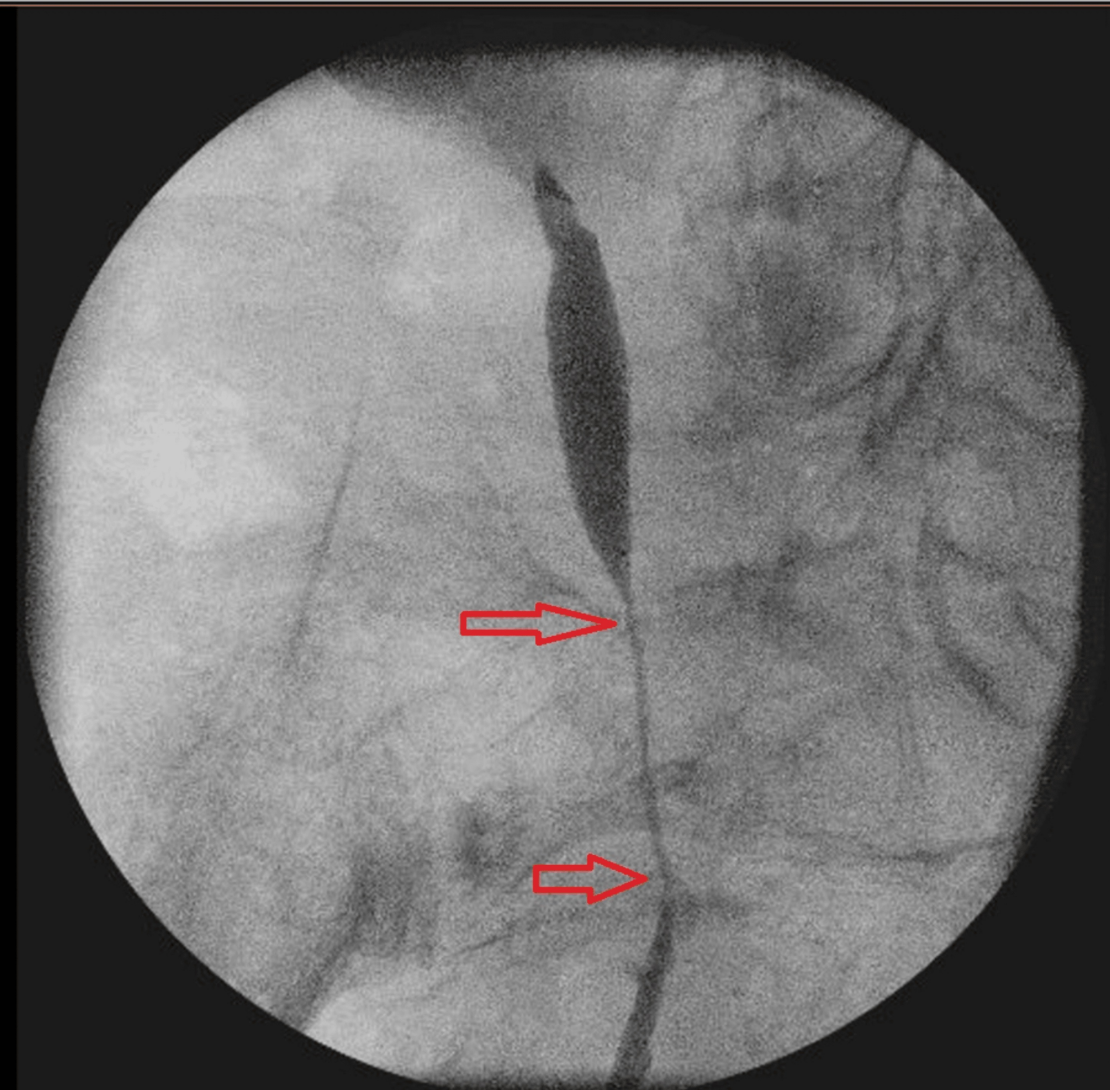
Right retrograde study showing a mid-ureteric stricture.

During the ureteric stent exchange procedure, we found that the bladder was filled with clots. After clot evacuation, a Sensor guidewire was successfully placed into the right kidney alongside the existing stent, which was then removed. The Sensor guidewire was subsequently removed to perform retrograde ureteropyelography, which was completed without complication. The retrograde study demonstrated a long mid-ureteric stricture measuring approximately 5 cm (Figure 1).

**Figure 2:**
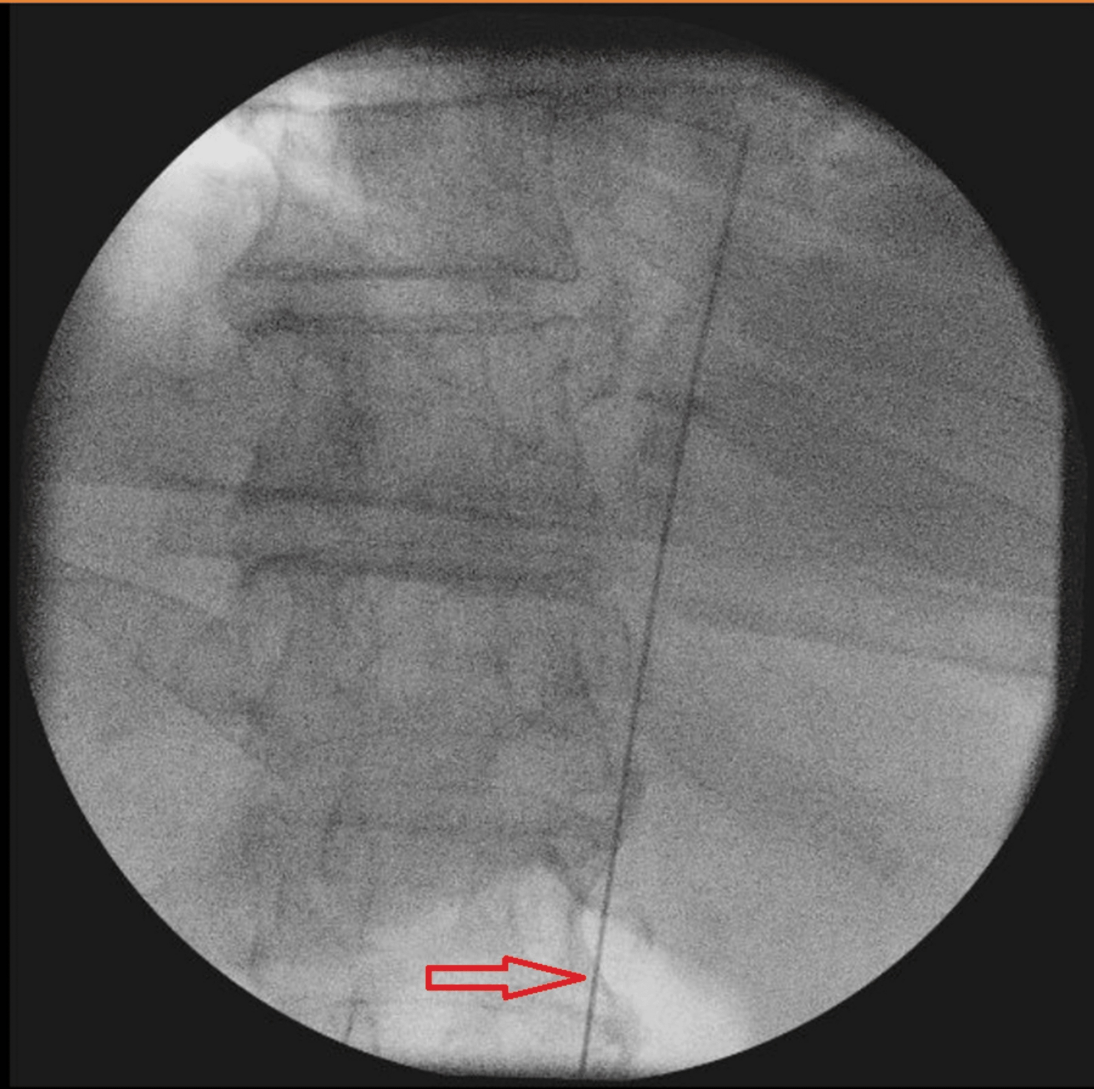
Image obtained with an image intensifier showing the guidewire passing from the right to the left side, crossing the spine.

Following this, a subsequent attempt to pass a guidewire through the ureteric catheter revealed an anatomically incorrect position, with the guidewire crossing the spine (Figure 2).

**Figure 3:**
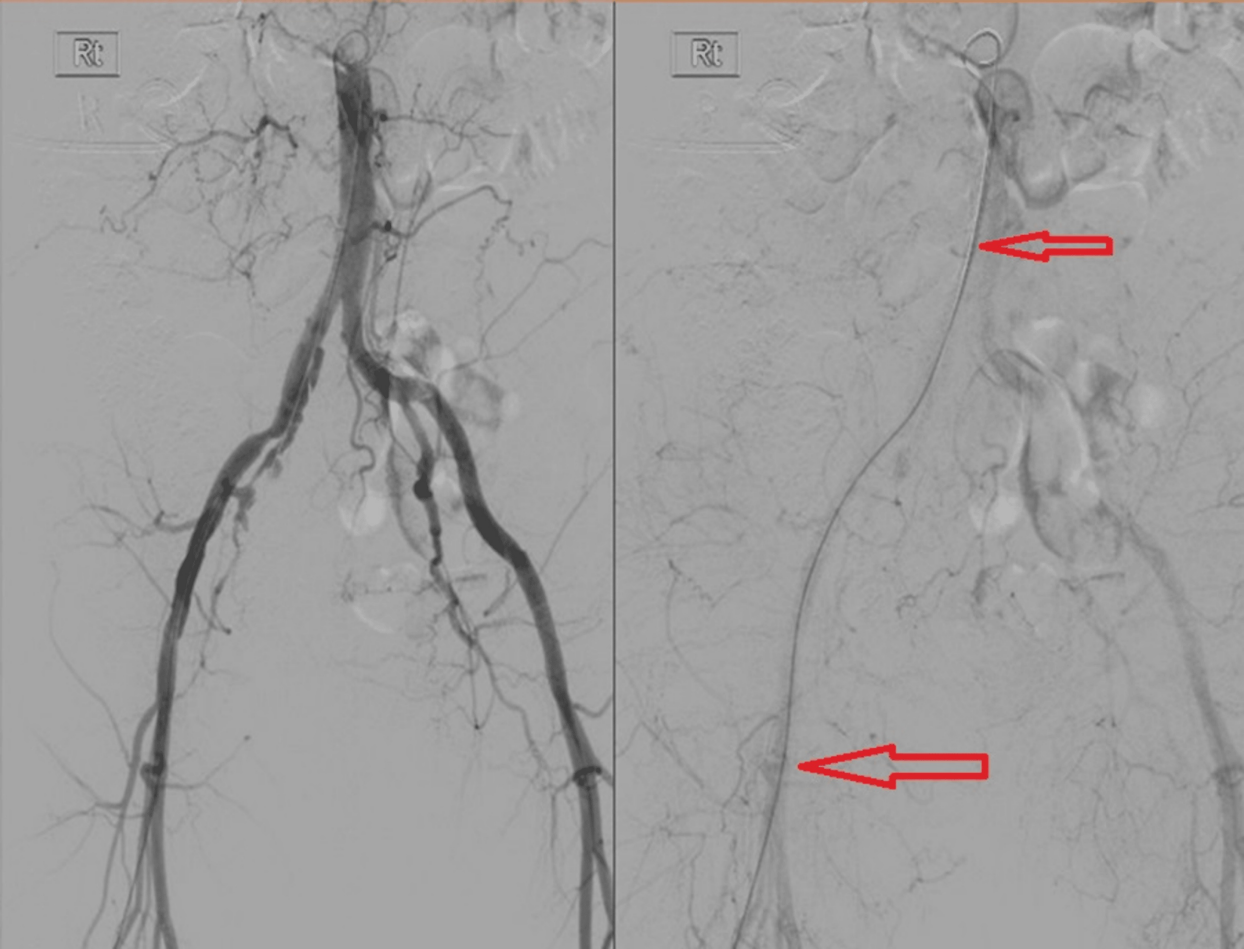
Angiography showing the guidewire passing through the right common iliac artery into the aorta (image provided by the vascular team).

Following this, the patient’s systolic blood pressure dropped to 60 mmHg. We strongly suspected that the guidewire had entered the vascular system. An urgent consultation was held with the interventional radiology (IR) and vascular surgery teams. It was decided to leave the guidewire in situ, and the patient was transferred, while intubated, to the IR suite. Vascular access to the right common iliac artery was obtained via the femoral artery, and angiography was performed. During angiography, it was confirmed that the guidewire was in the aorta after passing through the right common iliac artery (Figure 3).

**Figure 4:**
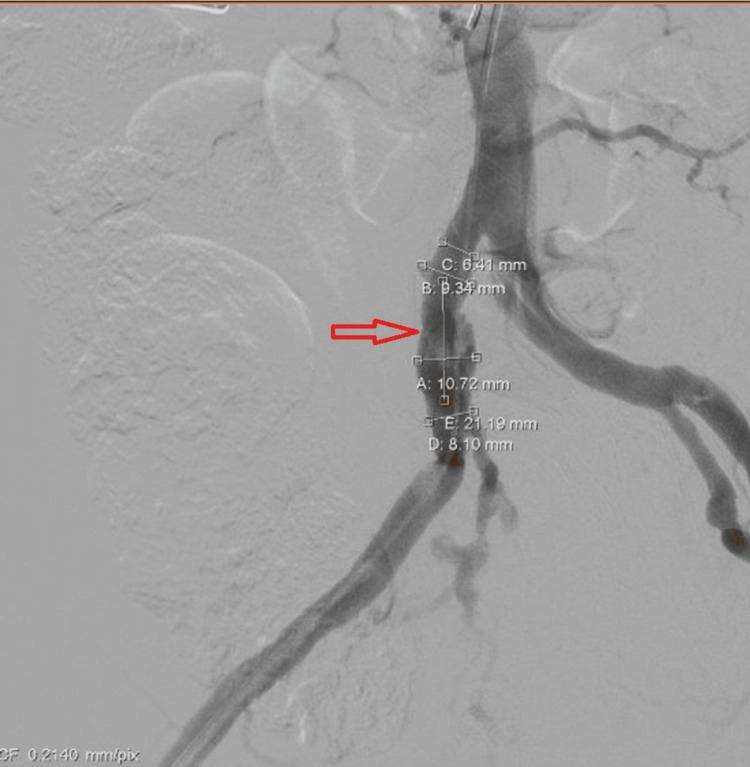
Fluoroscopy image following arterial stent graft insertion.

Multiple attempts to cannulate the right internal iliac artery were unsuccessful, likely because of stenosis of the ostium of the internal iliac artery or a dissection flap extending from the right common iliac artery to the internal iliac artery ostium. Following discussion with the vascular surgeons, a decision was made to deploy a covered stent graft in the right common iliac artery. A 10 × 37 mm covered vascular stent was successfully deployed in the right common iliac artery, and the Sensor guidewire within the right ureter was removed. Post-stent angiography demonstrated good stent position, with no contrast extravasation or evidence of bleeding (Figure 4). The patient was transferred to the intensive therapy unit (ITU) for careful monitoring and resuscitation if needed. No bleeding or complications were observed during her ITU stay, and her systolic blood pressure remained >90 mmHg. She was therefore transferred to the ward after two days.

## Discussion

Radiotherapy exerts its therapeutic effect primarily through ionizing radiation-induced DNA damage, particularly double-strand breaks, leading to apoptosis, mitotic catastrophe, and vascular injury in rapidly proliferating cells [4]. In the urinary bladder, radiation may induce acute and chronic inflammatory changes characterized by urothelial edema, vascular ischemia, fibrosis, and reduced compliance, ultimately resulting in radiation cystitis and lower urinary tract dysfunction. These pathological effects are mediated by oxidative stress, endothelial injury, and progressive obliterative endarteritis affecting the transitional epithelium and submucosal tissues [5]. Given that the ureter is similarly lined by transitional (urothelial) epithelium and has a delicate vascular supply, comparable radiation-induced injury may occur, including ureteritis, fibrosis, ischemic strictures, and impaired peristalsis. Although ureteric complications are less common than bladder toxicity because of the ureter’s smaller radiation exposure volume, chronic fibrosis and stenosis are well-recognized delayed sequelae following pelvic irradiation [6]. The shared histological characteristics of the bladder and ureter support a common susceptibility to radiation-related urothelial and stromal damage.

The exact pathogenesis of UIAF is not fully understood but is thought to be related to underlying risk factors such as prior irradiation and ureteric stents. Any insult to the integrity of the ureter can result in necrosis and inflammation, which may be further exacerbated by the pulsatile pressure of the adjacent iliac artery on the ureter. Therefore, knowledge of these risk factors is essential for risk stratification of patients presenting with recurrent hematuria and for maintaining a high index of clinical suspicion. Other risk factors include pelvic or genitourinary surgery, peripheral arterial disease, and aberrant vascular pathology [7]. In this case, the ureteric stent may have provided structural support to the ureteral wall adjacent to the iliac artery, and its removal may have unmasked or contributed to the formation of the fistula.

A review of the current literature on UIAF highlights the diagnostic challenges and the importance of maintaining a high index of clinical suspicion. CTA remains the primary imaging modality, and some studies suggest the value of provocative angiography, in which ureteric stent manipulation is performed during angiography to provoke bleeding. Vascular surgery teams should be informed and prepared to intervene should this result in major bleeding [8].

Treatment requires a multidisciplinary approach involving urologists, vascular surgeons, and interventional radiologists. In recent years, endovascular treatment has become the preferred approach, particularly in the emergency setting [9]. Endovascular treatment includes covered stent graft placement, whereas open surgical options include primary repair of the iliac artery with a patch or a Dacron graft. Other vascular interventions include embolization of the iliac artery [10]. These vascular interventions control bleeding (hematuria); however, the fistula may persist, and embolization may compromise blood supply to the lower limb. Endovascular and open surgical management may also involve arterial bypass procedures. These approaches may be combined with urinary diversion, nephrostomy, or nephrectomy. Transcatheter embolization, with or without subsequent surgical bypass, is another effective treatment option.

## Conclusions

UIAF is a rare cause of hematuria that requires a high index of clinical suspicion to facilitate diagnosis. CTA is the primary diagnostic modality and should ideally be performed during active hematuria. Management, particularly for iatrogenic cases, requires a coordinated multidisciplinary team approach to treatment and follow-up. As UIAF often presents in the emergency setting, increasing awareness and maintaining a high index of clinical suspicion may reduce associated morbidity and mortality.
